# Supplementary material for: Glucose-Sensing Carbohydrate Response Element-Binding Protein in the Pathogenesis of Diabetic Retinopathy
Source: Cells. 2025 Jan 13;14(2):107. doi: 10.3390/cells14020107 (PMC11763462; doi:10.3390/cells14020107)
Supplement: Supplementary file 1 [file cells-14-00107-s001.zip › fig. S3.pdf]

Upstream Regulator	Molecule Type	Activation z-score	p-value of overlap	Target Molecules in Dataset
GABA	chemical - endogenous mammalian	-2.5	0.00000285	ACADM,ALDH5A1,ATP6V1E1,DDX39B, GNGT1,HADH,PRKCSH,RAP1B,RPL18,RPL30,RPS18,RPS25,RPS26
AR	ligand-dependent nuclear receptor	-2.4	0.356	ACTR3,ALDH1A1,CEND1,PRKAR2A,TF,TJP1
8-bromo-cAMP	chemical reagent	-2.2	0.229	ALDH3A2,ASS1,CRYAA/LOC102724652,EZR,MYH9
FMR1	translation regulator	-2.2	3.19E-17	ATP5IF1,ATP6V1E1,CEND1,EEF2,RPL10A,RPL11, RPL14,RPL18,RPL18A,RPL3,RPL30,RPL7A,RPL8,RPS18,RPS20,RPS25,RPS26, RPS4Y1,SYN1,TF
JUN	transcription regulator	-2.2	0.000115	ANXA1,CRYAA/LOC102724652,DARS1,EEF2,EZR,HK2,HNRNPA2B1,HNRNPU, MYH9,NRCAM,TF,TXN
CST5	other	-2.1	0.000329	EEF1D,EZR,HNRNPA2B1,HNRNPU,NCL,RTN3,SLC1A3,TXN
butyric acid	chemical - endogenous mammalian	-2.1	0.178	ALDH1A1,ANXA1,MYH14,NCL,PRKAR2A
VDR	transcription regulator	-2.0	0.0957	CLIP1,HK2,TJP1,TXN
medroxyprogesterone acetate	chemical drug	-2.0	0.357	ALDH3A2,ASS1,EZR,MYH9
PML	transcription regulator	-2.0	0.0199	ACADM,CACYBP,SUMO2,TXN
torin1	chemical reagent	-2.0	4.14E-15	ATP5PB,ATP6V1E1,EEF1D,EEF2,HK2,RPL10A,RPL11,RPL14,RPL18,RPL3,RPL30, RPL7A,RPL8,RPS18,RPS20,RPS25,TPT1
5-fluorouracil	chemical drug	-1.8	4.51E-08	ABAT,ATP5PB,EEF2,GTF2I,HNRNPA2B1,HNRNPAB,ILF2,NACA,NCL,RPL10A,RPL11, RPL18,RPL30,RPL7A,RPS4Y1
LARP1	translation regulator	-1.6	1.61E-16	EEF1D,EEF2,RPL10A,RPL11,RPL14,RPL18,RPL3,RPL30,RPL7A,RPL8,RPS18,RPS20, RPS25,TPT1
sirolimus	chemical drug	-1.6	0.000000045	ACADM,ASS1,EEF2,FSCN1,HK2,HNRNPU,NCL,RPL10A,RPL18,RPL18A,RPL3,RPL30, RPL7A,RPL8,RPS18,RPS20,RPS26,RPS4Y1,TJP1
PAX3-FOXO1	fusion gene/product	-1.4	0.00242	ABAT,ANXA1,DNAJA2,EEF2,MYH9,NRCAM,RAB5C,TCP1
PTEN	phosphatase	-1.4	0.00417	ANXA1,CA2,CLTA,GPHN,GPM6A,GTF2I,HADH,PLEC,PLXNB2,PPM1A,RAB5A,RAB5C
RICTOR	other	-1.4	4.19E-11	ATP5PB,ATP6V1E1,ATP6V1H,HK2,PSMD1,RPL10A,RPL11,RPL14,RPL18,RPL30, RPL7A,RPL8,RPS18,RPS26,Rps3a1,RPS4Y1
aflatoxin B1	chemical - endogenous	-1.3	0.036	ALDH1A1,CA2,CRYAB,EZR,NRCAM

	non-mammalian			
TP73	transcription regulator	-1.3	0.0387	CRYAB,Elob,HAGH,LASP1,MYH9,PLEC,UBE2V1
MYOD1	transcription regulator	-1.3	0.00104	ASS1,CRYAB,ENO3,HADH,RPL7A,TJP1,TTN
SRF	transcription regulator	-1.2	0.0102	ACTR3,EZR,GPM6A,MYH9,TJP2,TTN,WDR1
benzo(a)pyrene	chemical toxicant	-1.1	0.0133	ANXA1,GNAO1,HK2,NBEA,TF
ST1926	chemical drug	-1.0	0.000000757	AP2S1,EEF2,ILF2,NACA,RPL18A,RPL8,RPS26,TPT1,TXN
nicotinamide-beta-riboside	chemical - endogenous mammalian	-1.0	0.000394	ALDH3A2,ALDH5A1,HADH,POR
NAMPT	cytokine	-1.0	0.00464	ALDH3A2,ALDH5A1,HADH,POR
tanespimycin	chemical drug	-1.0	0.00928	HNRNPA2B1,HNRNPAB,RPL30,SYN1
CTNNB1	transcription regulator	-0.8	4.69E-10	ACTR3,ALDH1A1,ALDH3A2,ANXA1,CTNND2,ENO3,Ewsr1,GNAO1,HK2,NRCAM, PRKCSH,RPL10A,RPL11,RPL14,RPL18,RPL18A,RPL3,RPL30,RPL7A,RPL8,RPS18,RPS20, RPS25,RPS26,Rps3a1,RPS4Y1,TJP1
EGR2	transcription regulator	-0.8	0.00924	ASS1,NCL,Ptms,SLC1A3,TJP2,TOP2B
CD 437	chemical drug	-0.7	0.0000414	AP2S1,EEF2,FSCN1,ILF2,NACA,RPL11,RPL8,TXN
topotecan	chemical drug	-0.7	0.00182	ASAP2,FUBP1,NBEA,NIPSNAP2,PPM1A,RAB10,RAB5A,SFPQ
AHR	ligand-dependent nuclear receptor	-0.7	0.0341	ALDH3A2,ALDH5A1,CRYAB,DMXL2,Gm21596/Hmgb1,HK2,MYH9,TXN
ESRRG	ligand-dependent nuclear receptor	-0.7	0.00709	ACADM,HADH,HK2,TTN
NFE2L2	transcription regulator	-0.6	0.0266	DDX39B,HK2,PSMD1,PSMD5,RPL18,Snrpa (includes others),TJP1,TXN
calcitriol	chemical drug	-0.5	0.026	CA2,HAGH,HK2,Hmgb3,POR,TF,TJP1,TJP2,TOP2B,TXN
L-triiodothyronine	chemical - endogenous mammalian	-0.5	0.0296	ALDH1A1,ASS1,KRT17,OPN1LW,POR,RGS9,TF,TTN
pidnarulex	chemical drug	-0.4	0.000214	ACTR3,ALDH1A1,ASS1,RPS4Y1,TPT1
ASPSCR1-TFE3	fusion gene/product	-0.4	0.00037	ATP6V1E1,CRYAB,EZR,PACSIN2,SNCB
TEAD1	transcription regulator	-0.4	0.00408	ACADM,ATP5PB,ETFA,HADH,TTN
HGF	growth factor	-0.4	0.0149	CRYAB,DDX3X,HK2,KRT17,NRCAM,RAB5A,SLK,TJP1

AKT1	kinase	-0.4	0.0425	ASS1,ATP6V1E1,CRYZ,EZR,Gm5938 (includes others)
VEGF (family)	group	-0.4	0.0179	CA2,CRYAB,DDX3X,HK2,NRCAM,RGS9,SLK,TJP1
HMGA1	transcription regulator	-0.4	0.00663	CNDP2,CRYAB,MYH9,RPL7A,TRIM28
ciprofloxacin	chemical drug	-0.4	0.00000773	ATP6V1E1,ATP6V1H,NRCAM,PLXNB2,RPL10A,RPL7A,RPS18
TYA-018	chemical reagent	-0.4	0.000286	ACADM,ATP5PB,ETFA,GPHN,HADH,MYH14,TTN
Z-LLL-CHO	chemical - protease inhibitor	-0.4	0.00019	CRYAA/LOC102724652,CRYAB,CTNND2,HNRNPA2B1,PPM1A,PSMD1,RAB10, TJP1,TRIM28,TXN
GLI1	transcription regulator	-0.3	0.0226	ANXA1,EZR,HNRNPU,HUWE1,KRT17,PPM1A,RPL30,SLC1A3
PD98059	chemical - kinase inhibitor	-0.3	0.0121	ACTR3,ANXA1,CA2,GTF2I,HNRNPA2B1,HNRNPAB,PLEC,TF,TXN
NR1I2	ligand-dependent nuclear receptor	-0.3	0.0107	ALDH1A1,ALDH3A2,POR,TF,TJP1
methylprednisolone	chemical drug	-0.3	0.00665	ABAT,ALDH1A1,ALDH3A2,ANXA1,ASS1,ATP5IF1,HAGH,HSD17B10,NCL,POR
dexamethasone	chemical drug	-0.3	0.000461	ACADM,ACTR3,ALDH1A1,ANXA1,ASAP2,CA2,CHD4,CRYAA/LOC102724652, CRYAB,ETFA,EZR,FSCN1,GTF2I,HK2,HNRNPAB,ILF2,KPNB1,KRT17,PLEC,POR, PRKAR2A,Rps3a1,SLC1A3,SLC25A22,TF,TJP1,TPT1,TXN,WDR1
APP	other	-0.2	0.000201	ARF5,ATP6V1E1,CHD4,CLTA,GDI2,GNAO1,KPNB1,MADD,PDXP,PRKAR2A, RAB5A,SNCB,SYN1,TJP1,TOP2B,TPT1,TXN
EPO	cytokine	-0.2	0.0000291	CA2,ILF2,KPNB1,NACA,Rps3a1,RPS4Y1,TF,TJP1,TPT1,WDR1
ESR1	ligand-dependent nuclear receptor	-0.2	0.0189	AP2S1,ASS1,ATP6V1H,CA2,KPNB1,KRT17,MADD,NRCAM,POR,RAB5C, RPL18A,SFPQ,SPAG9,TJP1,TJP2,TNPO1
1,2-dithiole-3-thione	chemical reagent	-0.2	0.0019	DDX39B,PSMD1,PSMD5,RPL18,Snrpa (includes others),TXN
TP53	transcription regulator	-0.2	1.68E-11	ABAT,ACADM,ALDH1A1,ANXA1,ASS1,ATP5PB,CLASP1,CLTA,CRYAB,DCTN2, DDX3X,DNAJA2,Dync1i2,ENO3,ETFA,EZR,FUBP1,GPHN,GPM6A,HADH,HK2, HNRNPA2B1,KPNB1,LASP1,MYH9,PAICS,PLXNB2,PPM1A,PRKAR2A,PSMD1, RAB5A,RAB5C,RPS18,RPS20,RPS25,RPS26,SFPQ,SYN1,TJP1,TOP2B,TRIM28,TTN
INSULIN (family)	group	-0.2	0.0447	ACADM,CRYAB,DNAJA2,ETFA,GDI2,HK2,TCP1,TF,TJP1
oleic acid	chemical - endogenous mammalian	-0.2	0.0498	ABAT,ACADM,HADH,HK2

tretinoin	chemical drug	-0.1	0.0112	ACTR3,ALDH1A1,ALDH3A2,ANXA1,CA2,EEF1D,GNAO1,MYH9,NACA,NCL,POR,RPL11,RPL3,RPS20,RPS4Y1,SYN1,TF,TJP1,TOP2B,TPT1
RAF1	kinase	-0.1	0.0024	ANXA1,CA2,CRYAB,HNRNPA2B1,HNRNPAB,PLEC
PLX5622	chemical drug	-0.1	0.00686	ARF5,MAP1A,NCL,SNCB,SYN1,TJP1
beta-estradiol	chemical - endogenous mammalian	0.0	0.00055	ABAT,ACADM,ALDH3A2,ANXA1,ARF5,ASS1,ATP5PB,CA2,CRYAA/LOC102724652,CRYZ,EZR,FSCN1,GDI2,GTF2I,HADH,IPO5,KRT17,MGARP,MYH9,RAB5C,RGS9,RPL14,RPL3,RPL8,RPS4Y1,SLC1A3,SLK,SPAG9,TF,TJP1,TJP2,TTN,TXN
HNF4A	transcription regulator	0.0	0.000521	ACTR3,ALDH1A1,ALDH5A1,ATP6V1H,CLTA,CRYZ,DDX39B,DNAJA2,GATD3/LOC102724023,GTF2I,KPNB1,KRT17,NBEA,POR,PPME1,PSMD1,RAB10,RPL18,RPL18A,RPS18,RPS20,RPS25,SF3B1,SUMO2,TF,TXN,UBE2V1
phorbol esters	chemical - other	0.0	0.00253	ANXA1,CRYAA/LOC102724652,HK2,TXN
mir-210	microRNA	0.0	0.00341	ALDH5A1,LASP1,SMCHD1,TNPO1
COPS5	transcription regulator	0.0	0.00408	HNRNPU,KPNB1,NCL,PAICS,PLXNB2,RPL18
pirinixic acid	chemical toxicant	0.0	0.0228	ACADM,ALDH3A2,HADH,HSD17B10,PAICS,POR,TXN
EGFR	kinase	0.1	0.0296	ANXA1,CRYAB,HK2,HNRNPA2B1,KPNB1,KRT17,NCL,PLXNB2
ESRRA	transcription regulator	0.1	0.046	ACADM,ANXA1,HK2,MYH9,TTN
PPARA	ligand-dependent nuclear receptor	0.1	0.0121	ACADM,ALDH3A2,ASS1,HADH,HSD17B10,POR,TJP1,TXN
deoxycholate	chemical - endogenous mammalian	0.2	0.00131	ALDH1A1,HK2,SLC1A3,TJP1
tamoxifen	chemical drug	0.2	0.0269	ALDH3A2,ASS1,CA2,CRYZ,NRCAM,TXN
IL15	cytokine	0.2	0.035	ANXA1,ENO3,GDI2,HK2,HNRNPA2B1,TJP1,TJP2
TGFB2	growth factor	0.2	0.0392	ALDH1A1,CA2,HK2,SLC6A11
PPARGC1A	transcription regulator	0.2	0.0383	ABAT,ACADM,ALDH5A1,GNAO1,HK2,OPN1LW,PACSIN2
puromycin aminonucleoside	chemical reagent	0.2	0.00479	ACTR3,ANXA1,ETFA,HADH
KRAS	enzyme	0.2	0.0351	ACTR3,CRYAB,CRYZ,CTNND2,GTF2I,HK2,KPNB1,MADD,NCL,PI4KA,POR,TCP1
metribolone	chemical reagent	0.2	0.000365	ATP5PB,ATP6V1E1,CRYAB,CRYZ,CTNND2,ETFA,EZR,HADH,HK2,HSD17B10,POR
CD3 (complex)	complex	0.3	0.00211	ACTR3,ANXA1,CLIP1,GDI2,HNRNPA2B1,HUWE1,ILF2,NCL,PLEC,RPL30,UBE2V1

TCR (complex)	complex	0.3	0.00956	ATP5PB,HADH,HSD17B10,RPL10A,RPL18A,RPL3,RPL30
nicotine	chemical drug	0.4	0.0179	CA2,Gm21596/Hmgb1,GNAO1,ILF2,RAP1B
MYCL	transcription regulator	0.4	0.000192	DDX3X,GPHN,RPL10A,RPL11,RPL18
E. coli B4 lipopolysaccharide	chemical toxicant	0.4	0.0325	ANXA1,HK2,RAB10,RPS25,TF
CIP2A	other	0.4	0.0000827	CRYAB,ENO3,HK2,LASP1,NCL
GRIN2A	ion channel	0.4	0.00025	GNAO1,KPNB1,PLXNB2,PSPC1,SFPQ
HBEGF	growth factor	0.4	0.000485	GNAT2,GNGT1,HK2,MYH9,OPN1LW
SNCA	enzyme	0.4	0.01	ALDH1A1,DCTN2,DMXL2,Ewsr1,EZR,SLC6A11,SYN1,VAT1L
SP2509	chemical reagent	0.4	0.044	DDX3X,EZR,FUBP1,HNRNPA2B1,KPNB1
MTOR	kinase	0.5	0.000112	ACADM,ATP5PB,ETFA,GPHN,HK2,MADD,PGP,PRKAR2A,RPS18,UBE2O
geldanamycin	chemical drug	0.5	0.000417	ANXA1,CACYBP,DDX3X,GNAO1,PPM1A,RAB10,RAB5A,RAB5C
IL4	cytokine	0.5	0.0152	ANXA1,ASS1,CA2,CHD4,CLIP1,FSCN1,HK2,MYH9,NCL,PLEC,PRRC2C, RAB5A,RELCH,Snrpa (includes others),TJP2
FOS	transcription regulator	0.6	0.00434	CA2,CLIP1,DDX3X,EZR,HK2,HSD17B10,MYH9,RPS18,SUMO2,TXN
QKI	other	0.6	0.0000687	CLTA,FSCN1,GPHN,GPM6A,RAB5A,RAB5C
NFKBIA	transcription regulator	0.6	0.00796	CRYAB,DDX3X,FSCN1,GATD3/LOC102724023,Gm21596/Hmgb1,HK2,RPL8,RPS18
hydrogen peroxide	chemical - endogenous mammalian	0.6	0.0407	ANXA1,CRYAB,DNAJA2,EZR,PRKCSH,RPL7A,RTN3,TXN
KCNJ2	ion channel	0.7	0.00000103	CLASP1,CLIP1,Ktn1,MAP1A,MYEF2,MYH14,MYH9,PLEC
GnRH analog	biologic drug	0.7	0.00112	ATP6V1E1,CLTA,GATD3/LOC102724023,GDI2,PI4KA,PSMD1,RAB5C,UBE2V1
CD28	transmembrane receptor	0.7	0.00176	ACTR3,ANXA1,CLIP1,HK2,HUWE1,ILF2,NCL,RPL30
lipopolysaccharide	chemical drug	0.8	0.00173	ACADM,ALDH1A1,ANXA1,ASS1,CRYAB,DDX3X,EEF2,FSCN1,GDI2,Gm21596/ Hmgb1,GPM6A,HK2,IPO5,LASP1,MYH14,MYH9,NCL,NIPSNAP2,PI4KA,PPM1A, RAB10,RAB5A,Snrpa (includes others),SPAG9,SUMO2,TF,TJP1,TPT1,TXN,UBE2V1
FOXA1	transcription regulator	0.8	0.0182	ANXA1,HADH,HK2,MYH9,TF
CX3CR1	G-protein coupled receptor	0.8	0.000885	CLASP1,CRYAA/LOC102724652,CRYAB,SFPQ,SYN1,TF
forskolin	chemical toxicant	0.8	0.0479	CRYAA/LOC102724652,CRYAB,EZR,HK2,PRKAR2A,RAB5A,RAB5C,RPS20,TJP1

TNF	cytokine	0.9	0.0312	ACADM,ANXA1,ASS1,CA2,CLASP1,CRYAB,FSCN1,GATD3/LOC102724023, GNAT2,GNGT1,HK2,MYH9,OPN1LW,PLXNB2,RGS9,SLC1A3,TF,TJP1,TJP2,TPT1,TXN
HIF1A	transcription regulator	0.9	0.0341	ENO3,FSCN1,HK2,HUWE1,MYH9,TJP1,TTN,TXN
TSC2	other	0.9	0.021	ANXA1,CRYAB,HK2,PSMD1
gentamicin	chemical drug	1.0	0.000359	ABAT,ACADM,ALDH1A1,DNAJA2,GPM6A,HNRNPU,NCL,SFPQ,TJP1
CPT1B	enzyme	1.0	0.000371	ACADM,ATP6V1H,CA2,FUBP1,HADH,HK2,PRKAR2A
mono-(2-ethylhexyl)phthalate	chemical toxicant	1.0	0.0206	ACADM,ATP5PB,HADH,HK2
VEGFA	growth factor	1.0	0.0486	ACADM,ETFA,RAB5A,TJP1,TJP2
ADIPOR1	transmembrane receptor	1.1	0.00233	ACADM,Hmgb3,OPN1LW,PLEC
FGFR1	kinase	1.1	0.0195	HK2,PLXNB2,SFPQ,TXN
lactic acid	chemical - endogenous mammalian	1.1	0.00851	CACYBP,CEND1,HK2,NRCAM,TXN
TGFB1	growth factor	1.1	0.00584	ALDH3A2,ALDH5A1,ASS1,CHD4,ENO3,FSCN1,FUBP1,GATD3/LOC102724023, GNAO1,HADH,HK2,HNRNPAB,HSD17B10,KRT17,LASP1,MYH9,NBEA,PPM1A, PSMD1,RAB1A,TJP1,TJP2,VAT1L
BDNF	growth factor	1.2	0.0196	EEF1D,EEF2,FSCN1,GNAO1,PLXNB2,SYN1
RCN3	other	1.3	0.0000154	RPL11,RPL3,RPL8,RPS18,RPS25
fluoride	chemical - endogenous mammalian	1.3	0.000163	ACTR3,DDX39B,GNAO1,HNRNPA2B1,TOP2B
RHO	G-protein coupled receptor	1.4	0.00194	ACADM,GNAT2,GNGT1,HK2,RGS9,SF3B1
LH (complex)	complex	1.6	5.98E-14	ATP5IF1,EEF2,EZR,HK2,PRKAR2A,PSMD1,RAB1A,RAB5A,RAB5C,RPL10A, RPL18A,RPL3,RPL30,RPL8,RPS18,RPS20,RPS25,RPS26,Rps3a1,RPS4Y1
E2F1	transcription regulator	1.7	0.00721	ACTR3,CA2,CRYAB,DDX39B,NCL,RAB1A,SMARCC2,TOP2B,TRIM28
uranyl nitrate	chemical toxicant	1.7	2.73E-08	ATP5IF1,CRYZ,EEF2,GATD3/LOC102724023,HNRNPA2B1,RAB5C,RPL10A, RPS26,TPT1
miR-124-3p (and other miRNAs w/seed AAGGCAC)	mature microRNA	2.0	0.0626	HADH,MTPN,MYH9,TJP2
HSP90B1	other	2.0	0.000513	ARF5,RAB10,RPL30,RPS20
LEP	growth factor	2.0	0.219	ACADM,ASS1,ATP5PB,Gm21596/Hmgb1,SYN1

MYCN	transcription regulator	2.0	8.95E-10	ALDH1A1,EEF1D,EEF2,HK2,MYH9,NACA,NCL,RPL11,RPL18,RPL18A,RPL3, RPL30,RPL8,RPS20,RPS25,RPS26
3,5-dihydroxyphenylglycine	chemical reagent	2.2	3.2E-17	ACTR3,ALDH1A1,RPL10A,RPL11,RPL14,RPL18,RPL18A,RPL3,RPL30,RPL7A, RPL8,RPS18,RPS20,RPS25,RPS26,RPS4Y1,TPT1
AGN194204	chemical drug	2.2	0.0133	ASS1,CA2,ENO3,HK2,Hmgb3
MLXIPL/ChREBP	transcription regulator	2.3	4.09E-14	RPL10A,RPL11,RPL14,RPL18,RPL18A,RPL3,RPL30,RPL7A,RPL8,RPS18, RPS20,RPS25,RPS26,Rps3a1,RPS4Y1
MYC	transcription regulator	2.4	4.66E-14	ACADM,ACTR3,ADD1,ASS1,ATP5IF1,CRYAB,DDX39B,DDX3X,EEF2,EZR, GDI2,HK2,HNRNPA2B1,HNRNPAB,HNRNPU,KRT17,NCL,PAICS,RAB10, RPL10A,RPL11,RPL14,RPL18,RPL18A,RPL3,RPL30,RPL7A,RPL8,RPS18, RPS20,RPS25,RPS26,Rps3a1,RPS4Y1,SUMO2,TF,TXN
