# Supplementary material for: Glucose-Sensing Carbohydrate Response Element-Binding Protein in the Pathogenesis of Diabetic Retinopathy
Source: Cells. 2025 Jan 13;14(2):107. doi: 10.3390/cells14020107 (PMC11763462; doi:10.3390/cells14020107)
Supplement: Supplementary file 1 [file cells-14-00107-s001.zip › Supplemental Figure Legend 12.23.24.pdf]

Figure S1. ERG analysis was performed on caChREBPRP mice at three time points: 20 dB, 0 dB, and 10 dB. Statistical significance (*t*-test) was assessed across groups: NS- non-significant; p*<0.05, n=3-6 per group.

Figure S2. Quantitative spectrum counts for the proteomic analysis of caChREBP^{RP} retinas.

Fig. S3. Ingenuity pathways significantly changed by ChREBP overexpression in rods of caChREBP^{RP} mice. Upstream regulator MLXIPL/ChREBP had the highest positive Z score activating transcriptional program.

Figure S4. Quantitative spectrum counts for the proteomic analysis of the ARPE-19 cells overexpressing ChREBP.

Figure S5. A violin plot was generated using single-cell RNA-seq (scRNA-seq) data from the human retina, retinal pigment epithelium (RPE), and choroid, uploaded to the Spectacle database https://singlecell-eye.org/app/spectacle.This dataset was contributed by the Institute for Vision Research at the University of Iowa, based on studies conducted between 2019 and 2020.

Figure S6. A violin plot was generated using single-cell RNA-seq (scRNA-seq) data from the mouse retina uploaded to the Spectacle database https://singlecell-eye.org/app/spectacle/ . This dataset was generated by the study titled “Molecular characterization of foveal versus peripheral human retina by single-cell RNA sequencing” by AP Voigt, SS Whitmore, MJ Flamme-Wiese, MJ Riker, LA Wiley, BA Tucker, EM Stone, RF Mullins, TE Scheet pubishe din experimental eye Research in 2019.
