# Supplementary figures and images for: Glucose-Sensing Carbohydrate Response Element-Binding Protein in the Pathogenesis of Diabetic Retinopathy
Source: Cells. 2025 Jan 13;14(2):107. doi: 10.3390/cells14020107 (PMC11763462; doi:10.3390/cells14020107)

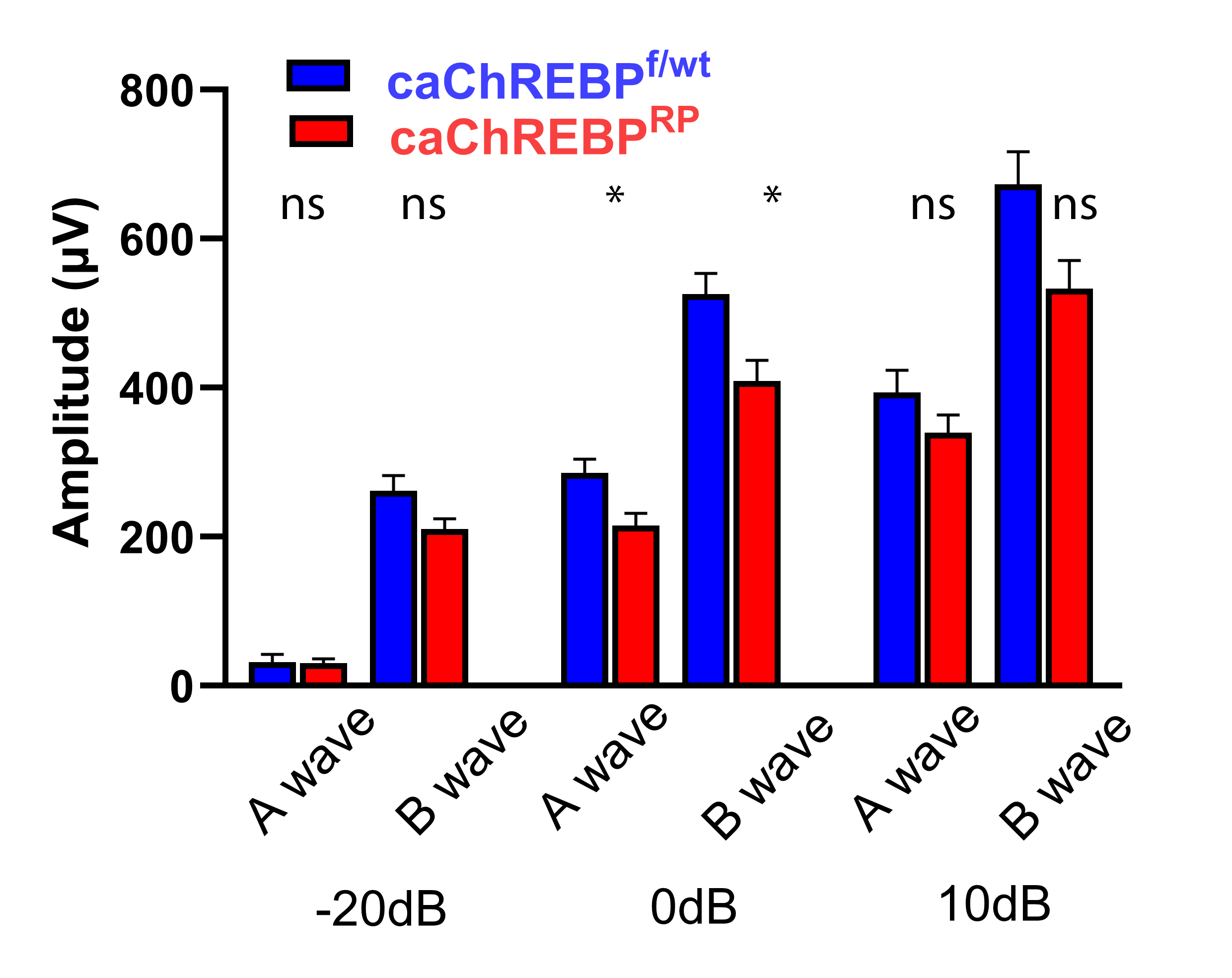

Supplement: Supplementary file 1 [file cells-14-00107-s001.zip › Fig. S1 12.23.24.tif]

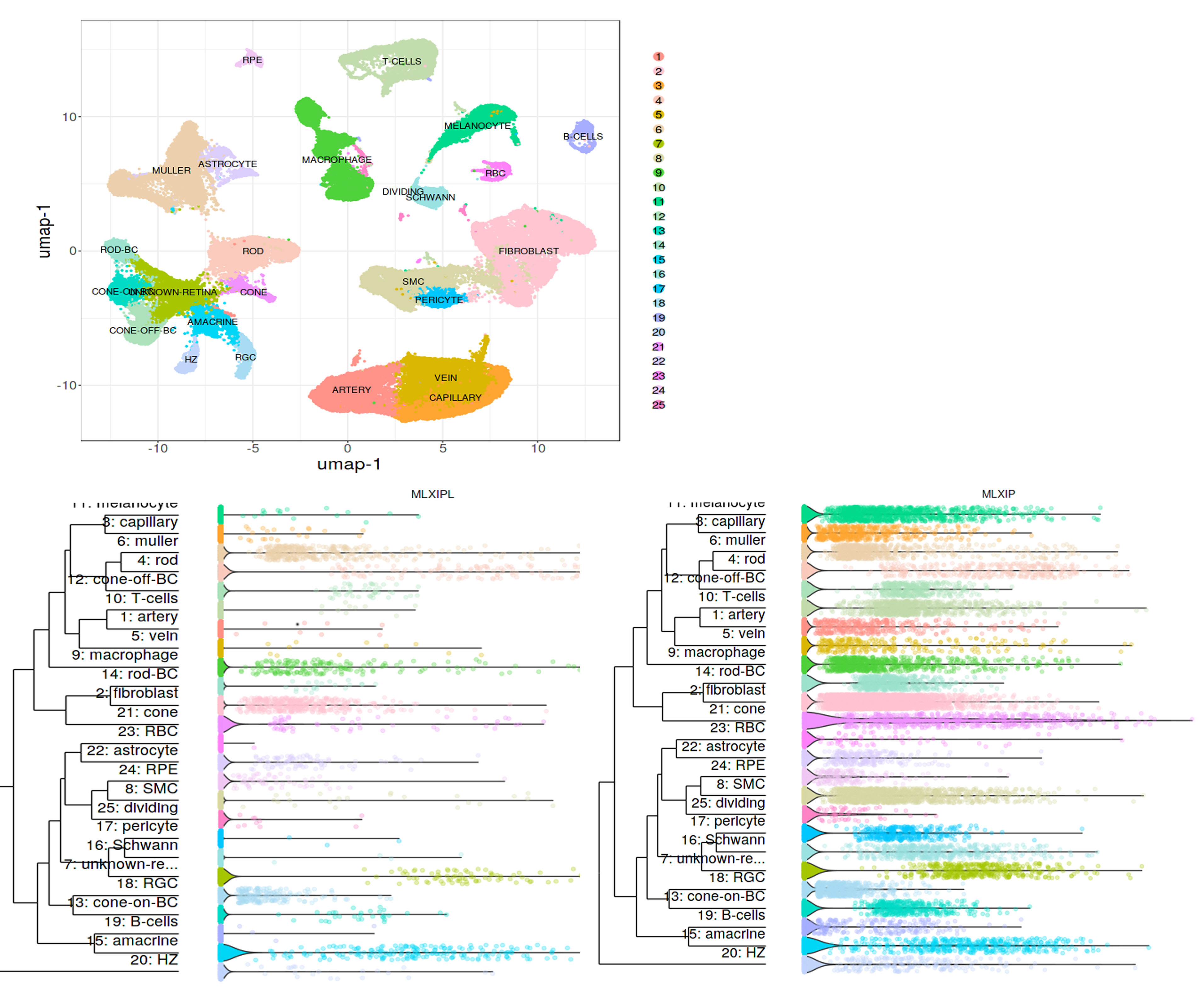

Supplement: Supplementary file 1 [file cells-14-00107-s001.zip › Figure S5 copy.jpg]

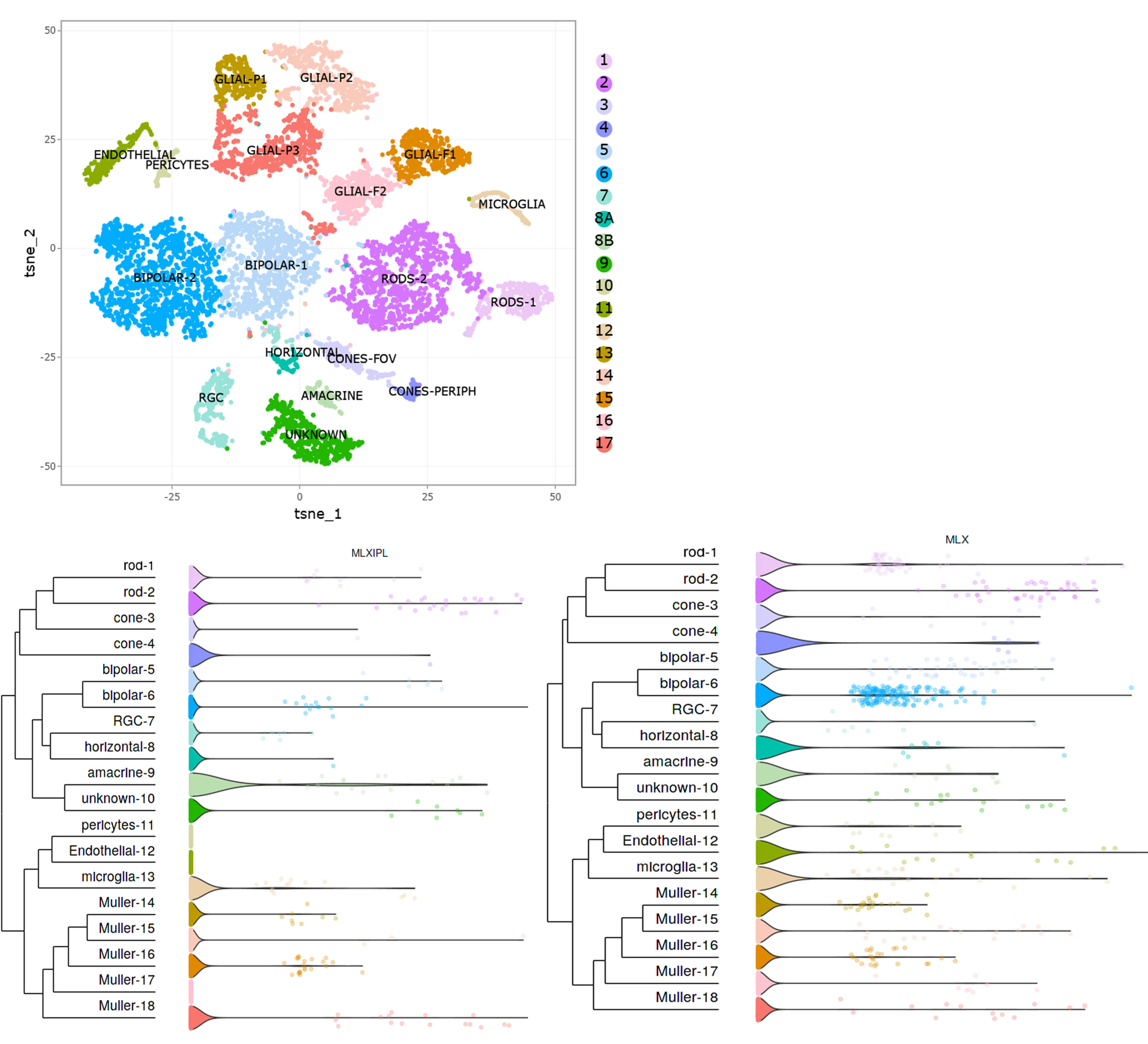

Supplement: Supplementary file 1 [file cells-14-00107-s001.zip › Figure S6 copy.jpg]
